# Supplementary material for: An explainable model of host genetic interactions linked to COVID-19 severity
Source: Commun Biol. 2022 Oct 26;5:1133. doi: 10.1038/s42003-022-04073-6 (PMC9606365; doi:10.1038/s42003-022-04073-6)
Supplement: Supplementary file 2 — Supplementary Information [file 42003_2022_4073_MOESM2_ESM.pdf]

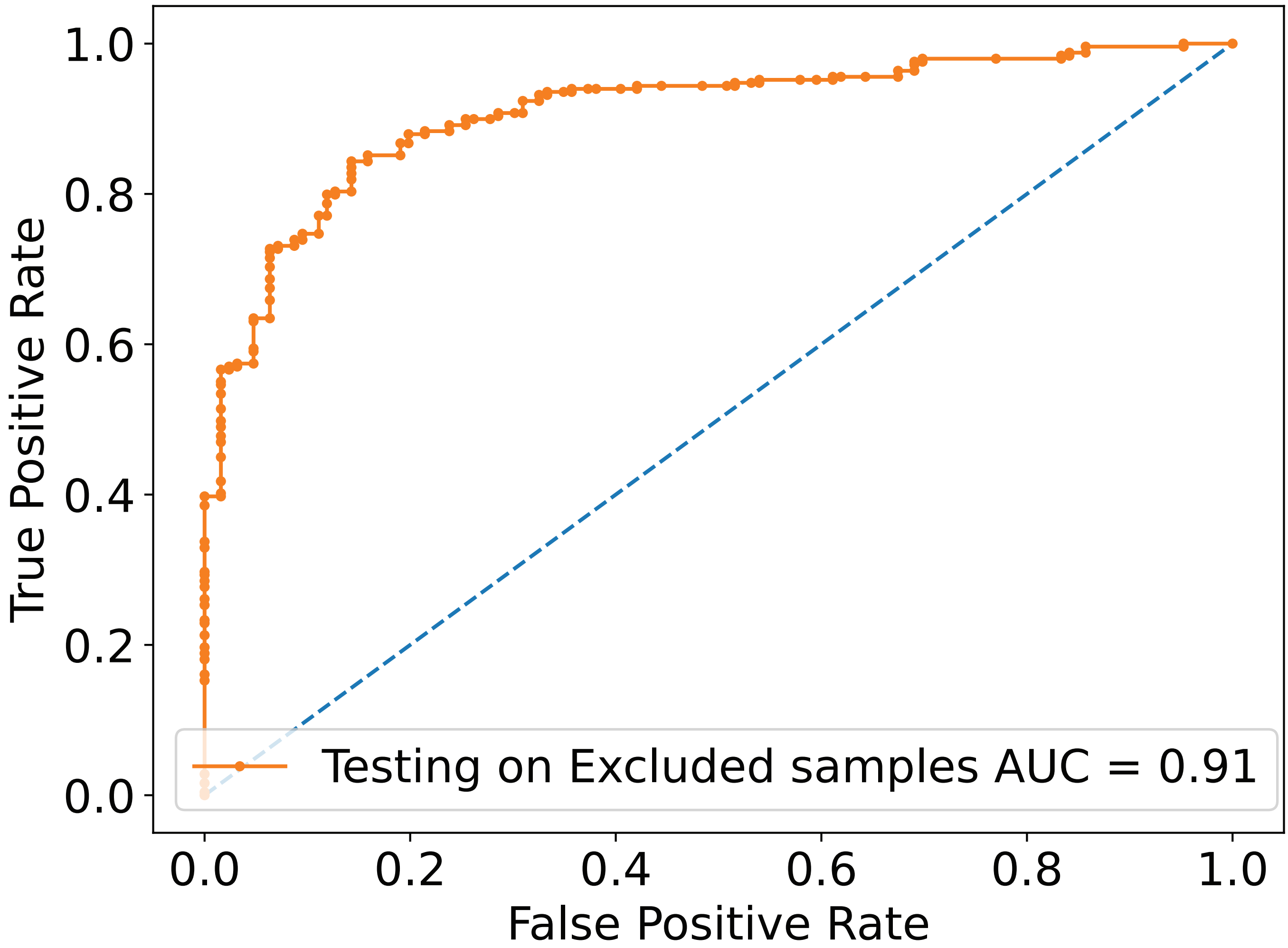


Performances of the predictors with 16 variants plus covariates (age and gender), on excluded samples from both training and testing set cohort (samples n=375).

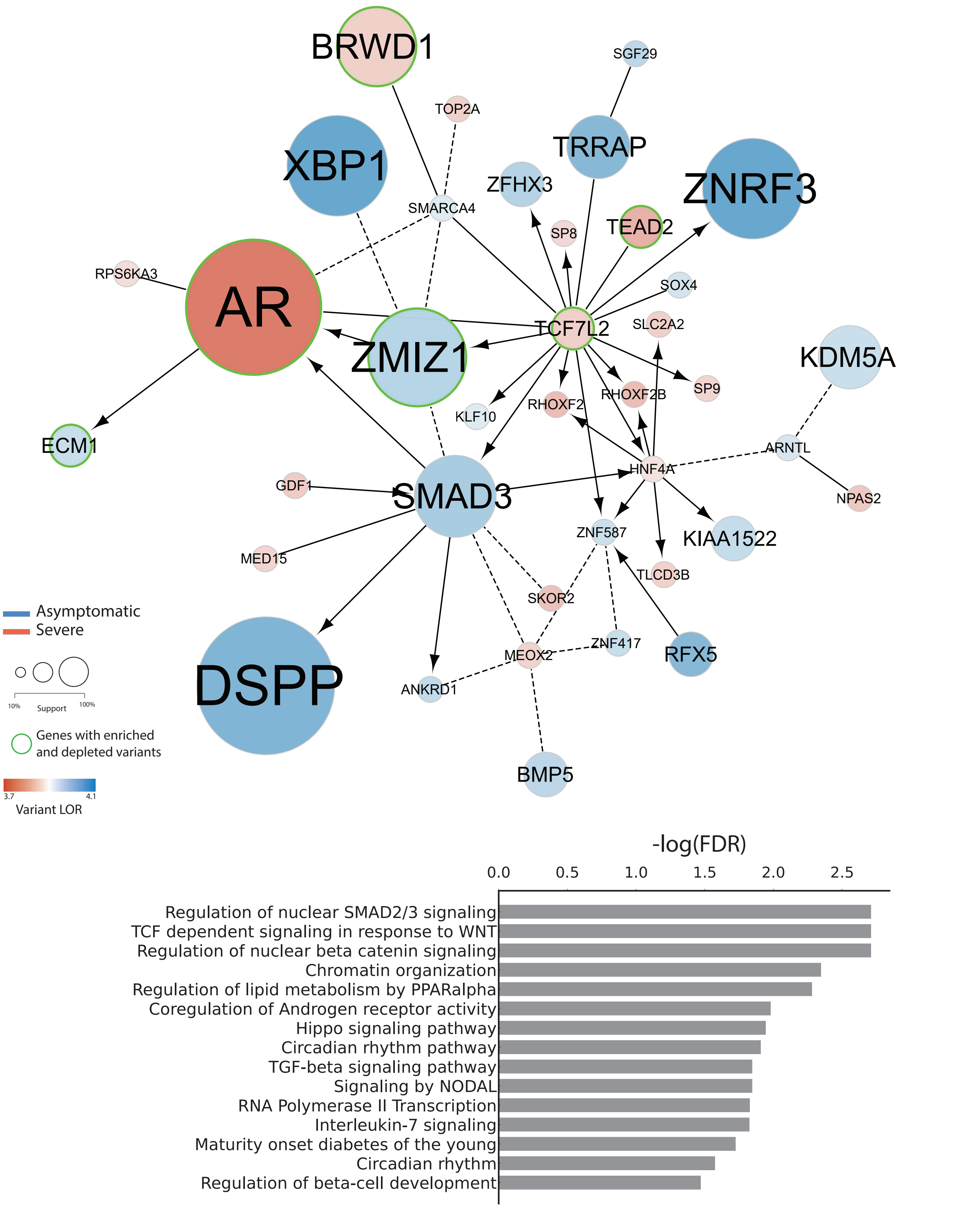


Top) Reactome FI network of genes of module 3 affected by variants with non-zero feature importance from XGBoost. Node diameter is proportional to the number of variants with non-zero coefficients in any tree based models. Node color is instead proportional to the LOR with the highest absolute value among the variants associated to a given gene ; bottom) barchart of the enriched processes within the module.

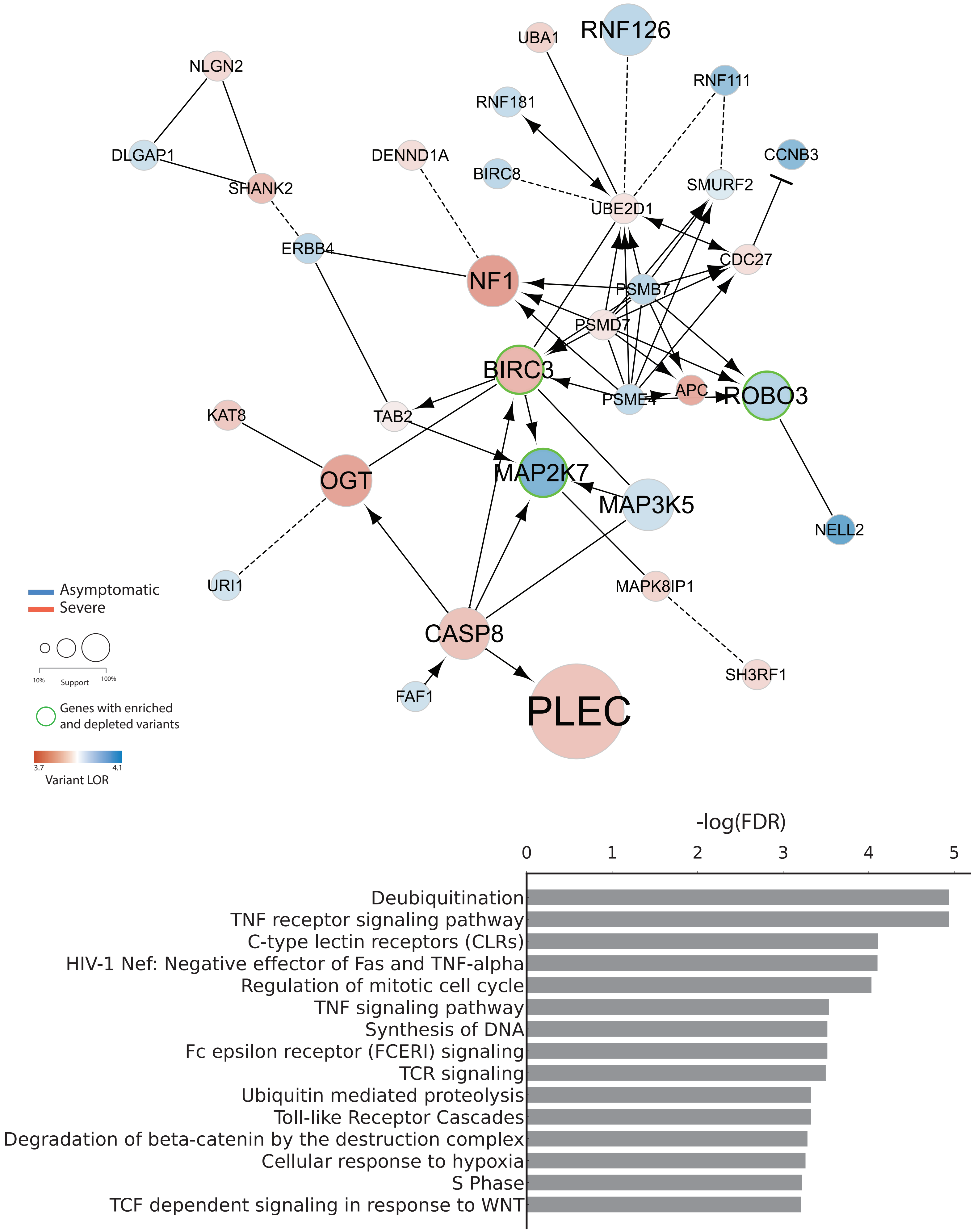


Top) Reactome FI network of genes of module 4 affected by variants with non-zero feature importance from XGBoost. Node diameter is proportional to the number of variants with non-zero coefficients in any tree based models. Node color is instead proportional to the LOR with the highest absolute value among the variants associated to a given gene; bottom) barchart of the enriched processes within the module.

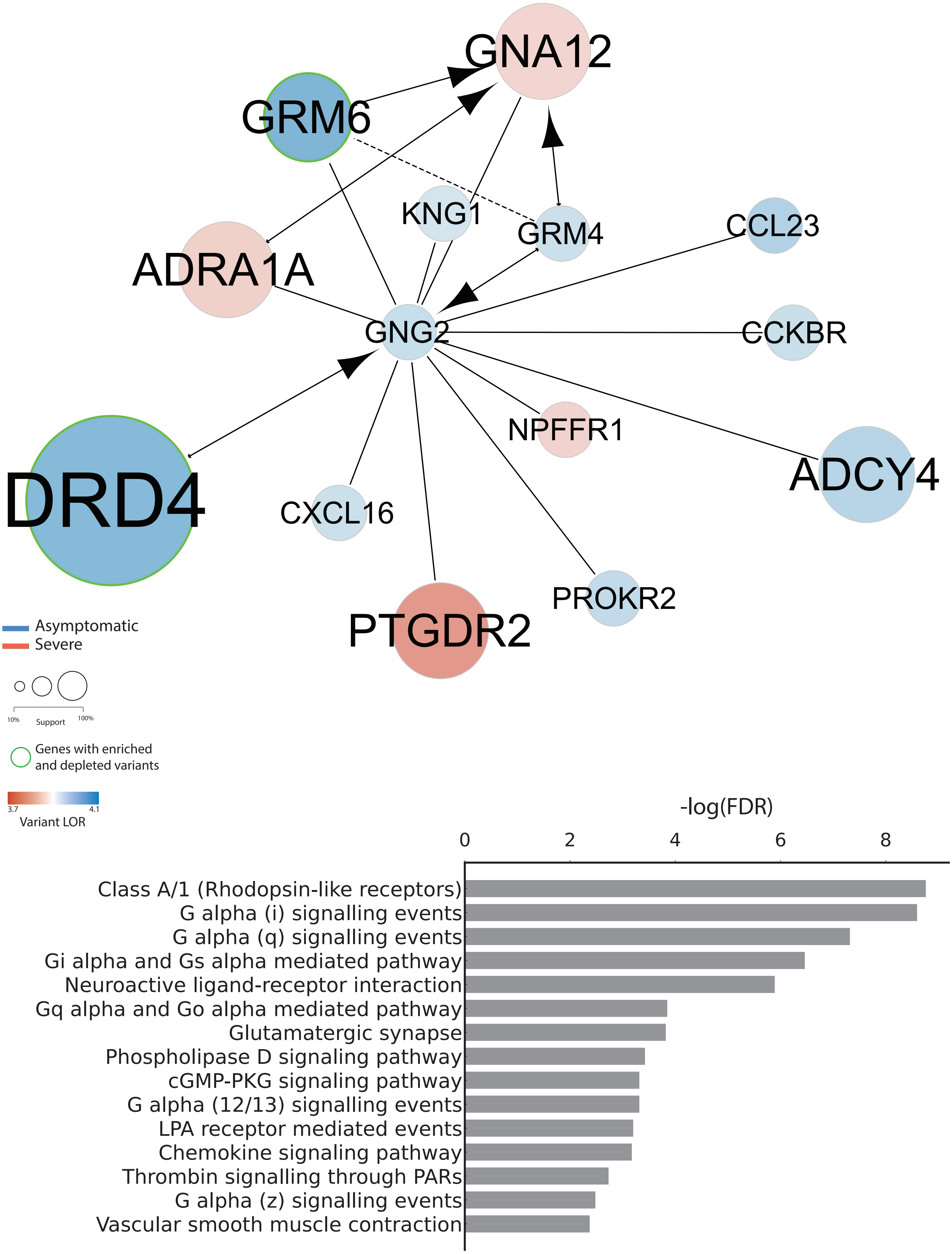


Top) Reactome FI network of genes of module 8 affected by variants with non-zero feature importance from XGBoost. Node diameter is proportional to the number of variants with non-zero coefficients in any tree based models. Node color is instead proportional to the LOR with the highest absolute value among the variants associated to a given gene; bottom) barchart of the enriched processes within the module.

a

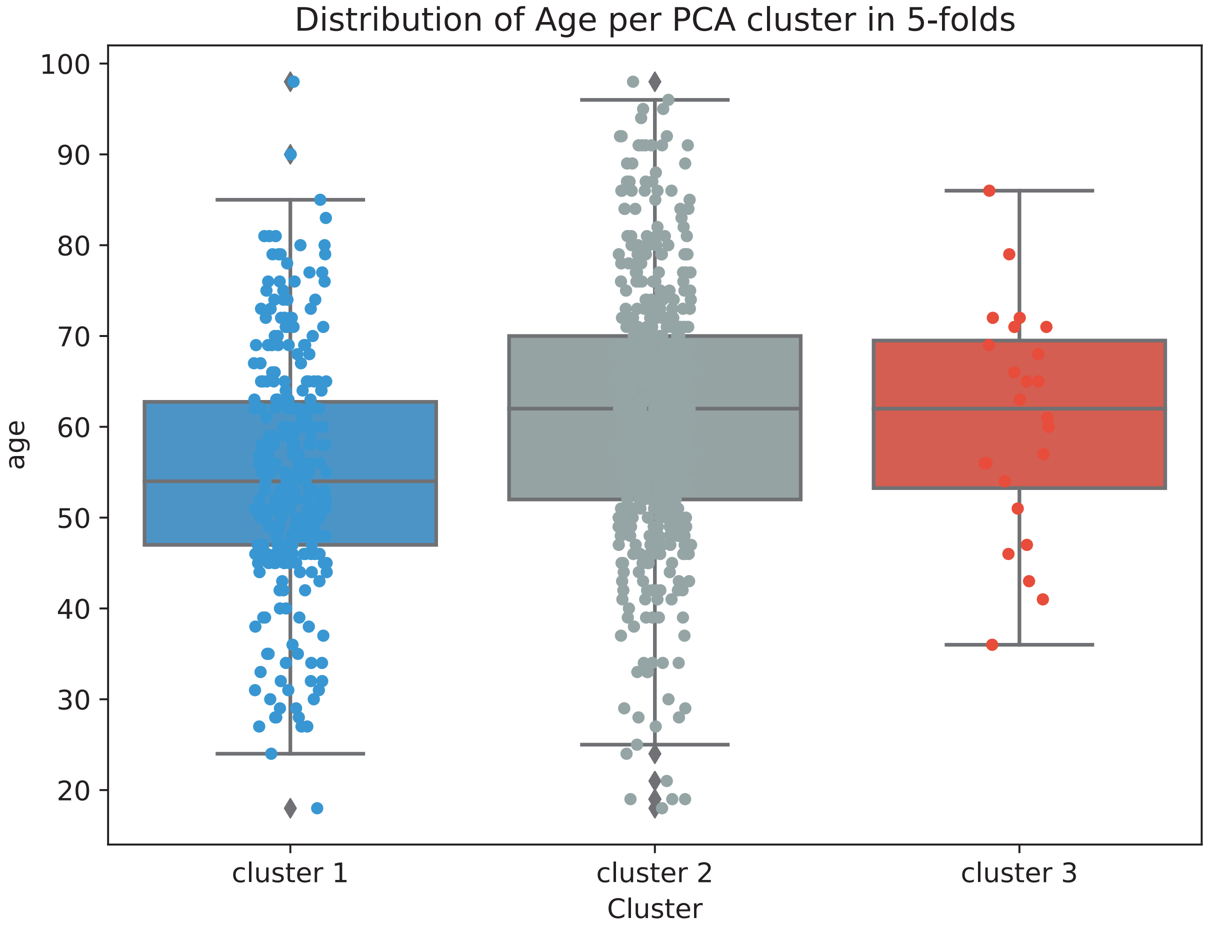


b

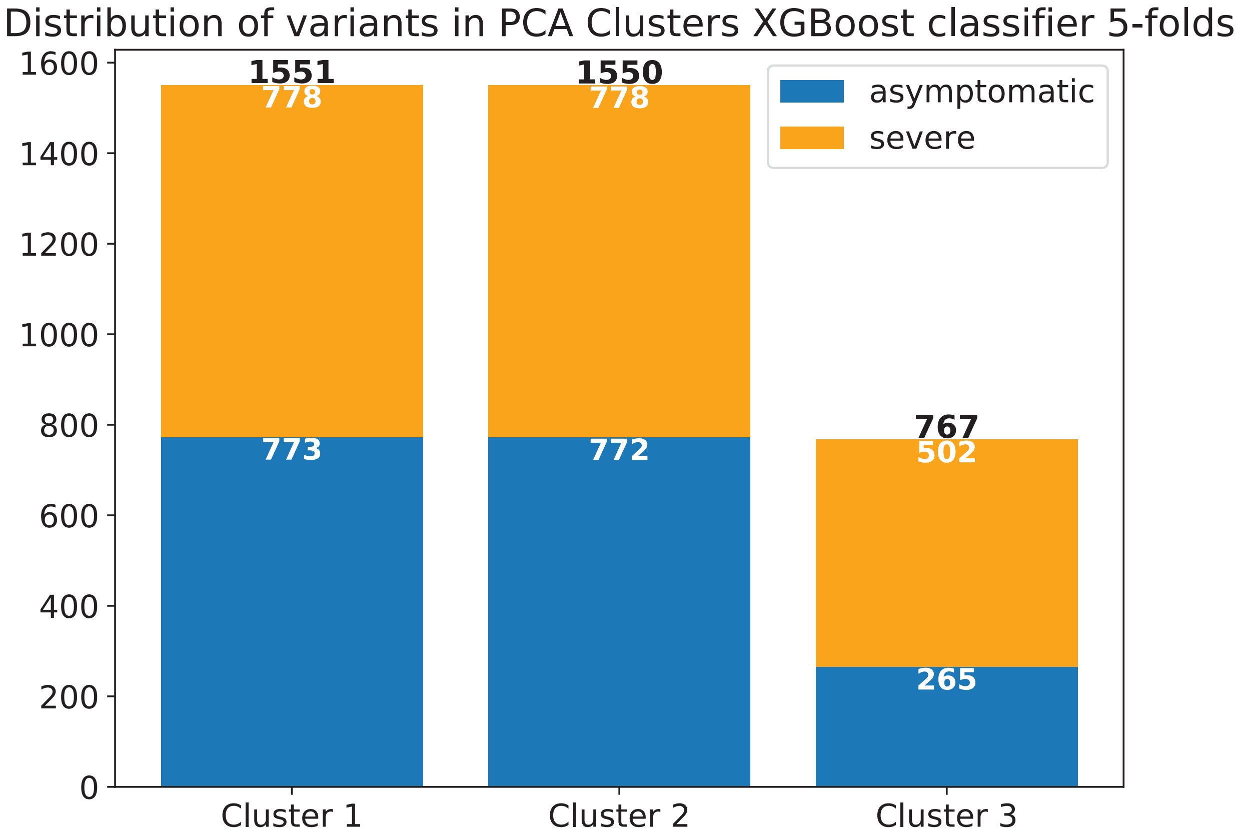


a) age distribution of the patients in the three clusters identified by PCA and k-means clustering considering non-zero importance variants in the cohort used for training (samples n=841). The horizontal line inside each box represents the median value, and the height (whiskers) of each of the boxes represent the standard error (variability). The dotted points above and below the individual box-and-whisker lines are potential outliers that are above or below the 25th percentile, and the 75th percentile; b) variant distribution in the three cluster.

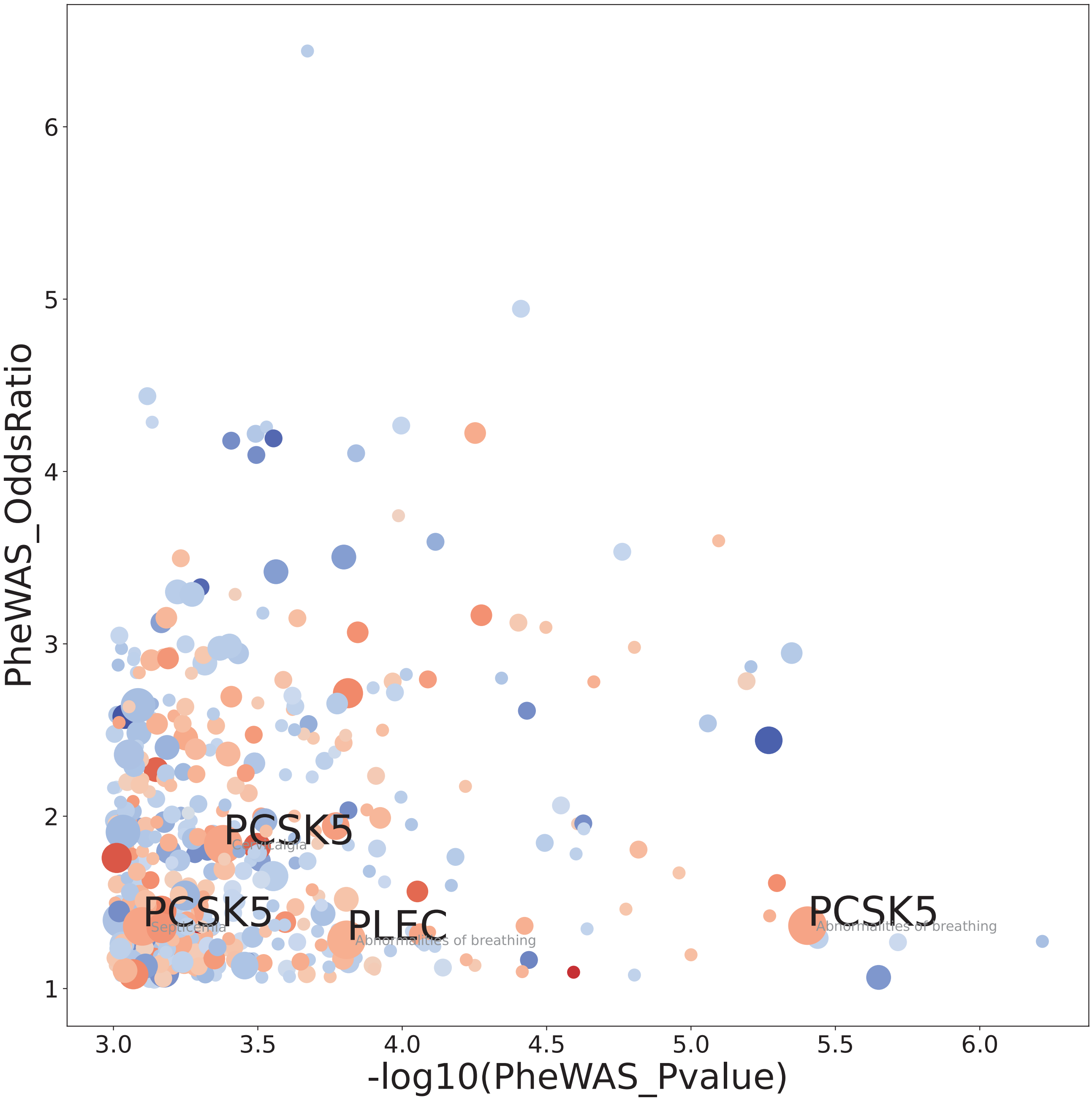


Scatter plot showing variant-specific traits associated within the "Phenotype" category.
Dot diameter is proportional to the model support for each variant. The color is proportional to the log-odds ratio of the variant in the two groups of the cohort. Labels are printed only associations with PheWAS Pvalue < 0.001 and PheWAS oddsratio > 2.5 or for variants having non-zero coefficients in at least one XGBoost model.

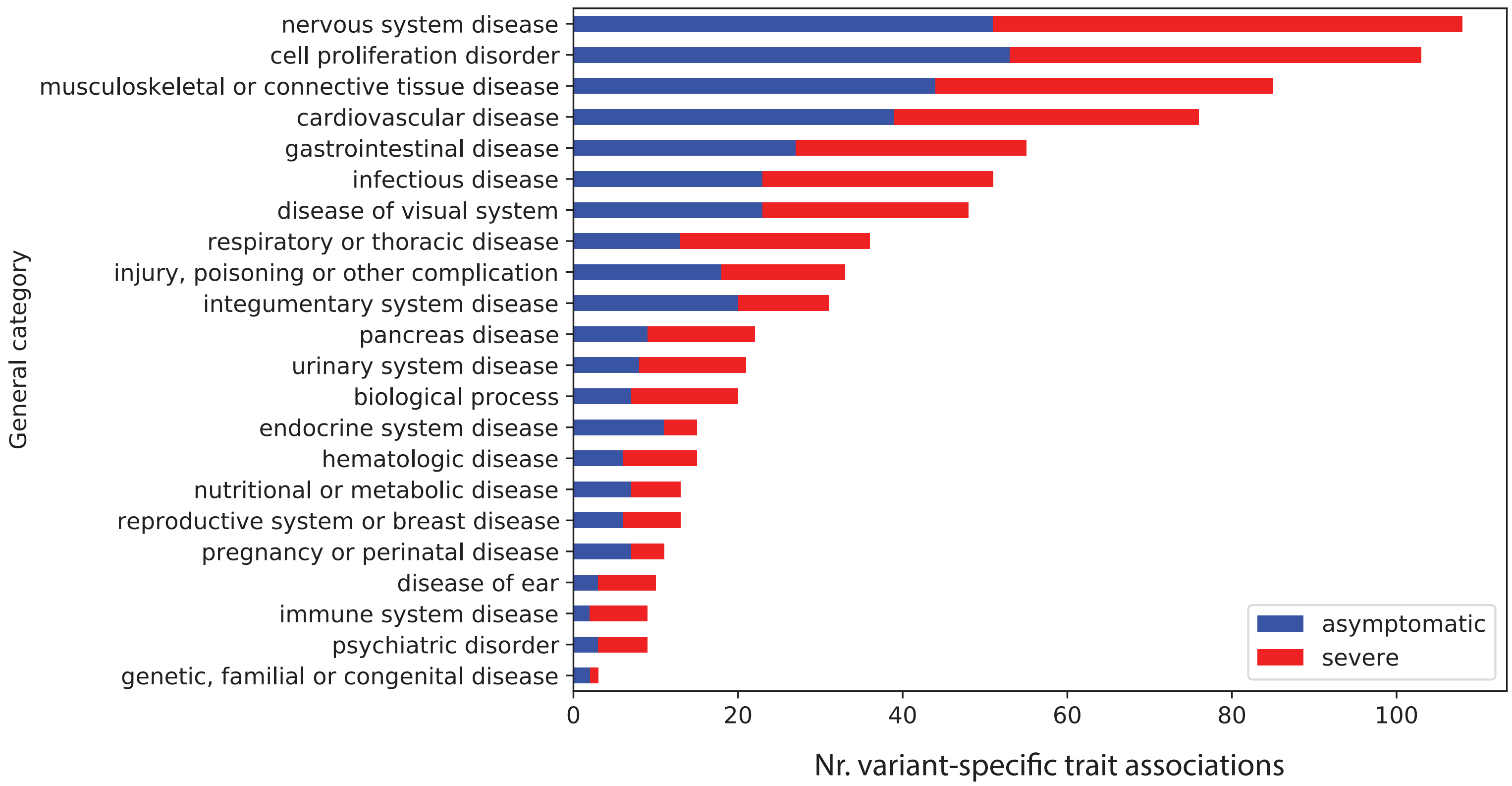


Barchart of the associations of traits to variants enriched in either severe (red) or asymptomatic (blue) patients for general categories.

| | *Severe* | *Asymptomatic* |
|---|---|---|
| | **Group 543** | **Group 0** |
| *Alt* | A | B |
| *Ref* | C | D |

Contingency table employed to perform log-odds ratio statistics for case(severe)-control (asymptomatic) associations.
