# Supplementary material for: An explainable model of host genetic interactions linked to COVID-19 severity
Source: Commun Biol. 2022 Oct 26;5:1133. doi: 10.1038/s42003-022-04073-6 (PMC9606365; doi:10.1038/s42003-022-04073-6)
Supplement: Supplementary file 3 — Description of Additional Supplementary Files [file 42003_2022_4073_MOESM3_ESM.pdf]

Description of Additional Supplementary Files

File name: Supplementary Data 1-13

Description:

Supplementary Data 1: The source data for Figure 2, 3, 4 and 5.

Supplementary Data 2-6, Supplementary Data 13: The source data for plots in Figure 2 and Figure S1.

Supplementary Data 7-9**:** The source data for plots and images in Figure 3 and Figure S2-4.

Supplementary Data 10-11**:** The source data for Figure 4.

Supplementary Data 12**:** The source data for Figure 5.
