# Supplementary material for: An explainable model of host genetic interactions linked to COVID-19 severity
Source: Commun Biol. 2022 Oct 26;5:1133. doi: 10.1038/s42003-022-04073-6 (PMC9606365; doi:10.1038/s42003-022-04073-6)
Supplement: Supplementary file 5 — Reporting Summary [file 42003_2022_4073_MOESM5_ESM.pdf]

nature portfolio

Corresponding author(s): DBPR and COMMSBIO-21-3545B

Last updated by author(s): Sep 2, 2022

Reporting Summary

Nature Portfolio wishes to improve the reproducibility of the work that we publish. This form provides structure for consistency and transparency in reporting. For further information on Nature Portfolio policies, see our Editorial Policies and the Editorial Policy Checklist.

Statistics

For all statistical analyses, confirm that the following items are present in the figure legend, table legend, main text, or Methods section.

n/a	Confirmed	
☐	☒	The exact sample size (n) for each experimental group/condition, given as a discrete number and unit of measurement
☐	☒	A statement on whether measurements were taken from distinct samples or whether the same sample was measured repeatedly
☐	☒	The statistical test(s) used AND whether they are one- or two-sided<br>*Only common tests should be described solely by name; describe more complex techniques in the Methods section.*
☐	☒	A description of all covariates tested
☐	☒	A description of any assumptions or corrections, such as tests of normality and adjustment for multiple comparisons
☐	☒	A full description of the statistical parameters including central tendency (e.g. means) or other basic estimates (e.g. regression coefficient) AND variation (e.g. standard deviation) or associated estimates of uncertainty (e.g. confidence intervals)
☐	☒	For null hypothesis testing, the test statistic (e.g. F, t, r) with confidence intervals, effect sizes, degrees of freedom and P value noted<br>*Give P values as exact values whenever suitable.*
☒	☐	For Bayesian analysis, information on the choice of priors and Markov chain Monte Carlo settings
☐	☒	For hierarchical and complex designs, identification of the appropriate level for tests and full reporting of outcomes
☐	☒	Estimates of effect sizes (e.g. Cohen's d, Pearson's r), indicating how they were calculated

Our web collection on statistics for biologists contains articles on many of the points above.

Software and code

Policy information about availability of computer code

Data collection	https://github.com/raimondilab/An-explainable-model-of-host-genetic-interactions-linked-to-Covid19-severity
Data analysis	https://github.com/raimondilab/An-explainable-model-of-host-genetic-interactions-linked-to-Covid19-severity

For manuscripts utilizing custom algorithms or software that are central to the research but not yet described in published literature, software must be made available to editors and reviewers. We strongly encourage code deposition in a community repository (e.g. GitHub). See the Nature Portfolio guidelines for submitting code & software for further information.

Data

Policy information about availability of data

All manuscripts must include a data availability statement. This statement should provide the following information, where applicable:

- Accession codes, unique identifiers, or web links for publicly available datasets
- A description of any restrictions on data availability
- For clinical datasets or third party data, please ensure that the statement adheres to our policy

https://github.com/raimondilab/An-explainable-model-of-host-genetic-interactions-linked-to-Covid19-severity
(https://clinicaltrials.gov/ct2/show/NCT04549831)

Field-specific reporting

Please select the one below that is the best fit for your research. If you are not sure, read the appropriate sections before making your selection.

☒ Life sciences ☐ Behavioural & social sciences ☐ Ecological, evolutionary & environmental sciences

For a reference copy of the document with all sections, see nature.com/documents/nr-reporting-summary-flat.pdf

Life sciences study design

All studies must disclose on these points even when the disclosure is negative.

Sample size

The observational study through which our study came into being, implored a non-probability sampling method to identify and enroll the samples of interest (individuals infected with SARS-CoV-2 and showing different clinical outcomes from asymptomatic to severely affected patients). The genetic and clinical data type we used for this study was gotten from the aforementioned observational study dataset coordinated by the GEN-COVID Multicenter Study group led by the University of Siena with an estimated enrollment of 2000 participants which constitutes the basis of the sampling size from which the genetic information was obtained (https://clinicaltrials.gov/ct2/show/NCT04549831). The observational model used to collect the Whole Exome Sequencing (WES) dataset of the patients enrolled is a Case-Control type with a time perspective. The official title of this process is called “the Identification of the Genetic Bases Determining COVID-19 Clinical Variability in the Italian Population”. Our study used the WES dataset and clinical covariates of 1982 sample size European descent patients provided to us by the GEN-COVID Multicenter Study group. Patients were classified according to the grading scheme by the World Health Organization (WHO). For the time being, the 2000 cohort of WES patients information has provided us with an enormous amount of genetic variants information (the WES dataset contained a total of 1.057M unique sample variants), however, as the study progresses more patient cohorts will be enrolled until information saturation is attained.

Data exclusions

We excluded patients' information whose severity grading was classified as 1, & 2. This is because we wanted to be purposeful to reduce noise signals from the filtered variants as much as possible, and be more stringent in identifying significant variants that might likely interplay with patients' host severity to SARS-CoV-2 infection and susceptibility to the coronavirus disease.

Replication

We made available all the codes we used to establish the findings of this study on our GitHub Bioinformatics Lab page repository (https://github.com/raimondilab/An-explainable-model-of-host-genetic-interactions-linked-to-Covid19-severity). It is also worthy to note that we carried out an external validation of our model with a 3000 follow-up cohort WES dataset to validate the reliability of the Host genetic predictor COVID-19 model aside from the internal validation we carried out with each 20% testing sets from the 5-fold CV splits.

Randomization

The patients phenotypic information on COVID-19 severity grading classification contained the following categories: 0=not hospitalized (a- or pauci-symptomatic); 1=hospitalized without respiratory support; 2=hospitalized O2 supplementation; 3=hospitalized CPAP-biPAP; 4= hospitalized intubated; 5=dead. We considered patients from more severe gradings, i.e. 3, 4, and 5, as severe cases grouped as 1, and asymptomatic patients from grade 0, as controls, grouped as 0. This constitutes a total of 1078 patients. We further refined the grading classification based on an ordinal logistic model which uses age as an input feature for sex-stratified patients (Fallerini, Picchiotti, et al., 2021) and we retained only those patients whose grading classification was concordant with the one adjusted by age. This yielded a final set of 841 samples for downstream analysis. The rationale for using the 841 sample size for further analyses was the virtue of their capacity to provide richly-textured information that is relevant to the identification of plausible genetic variants linked to COVID-19 severity in patients.

Blinding

The collaborator (GEN-COVID Multicenter Study group) adopt a double-blind, randomized clinical trial for this study, i.e., neither the participants know about the treatment group they are assigned to nor are we the researchers interacting with their genetic and clinical information. Patients' information such as sampling IDs were coded and concealed and by no means disclosed patients’ identity or private confidential information to the researchers carrying out the downstream analyses. The double-blinded trial approach adopted by the collaborator helped us to minimize unforeseen biasedness in the course of this study, developed a reliable host genetic predictor model, and insightful result findings to further shed light on the patients' COVID-19 severity.

Reporting for specific materials, systems and methods

We require information from authors about some types of materials, experimental systems and methods used in many studies. Here, indicate whether each material, system or method listed is relevant to your study. If you are not sure if a list item applies to your research, read the appropriate section before selecting a response.

Materials & experimental systems

n/a	Involved in the study	
☒	☐	Antibodies
☒	☐	Eukaryotic cell lines
☒	☐	Palaeontology and archaeology
☒	☐	Animals and other organisms
☐	☒	Human research participants
☐	☒	Clinical data
☐	☐	Dual use research of concern

Methods

n/a	Involved in the study	
☒	☐	ChIP-seq
☒	☐	Flow cytometry
☒	☐	MRI-based neuroimaging

Human research participants

Policy information about studies involving human research participants

Population characteristics	In this study, we considered only patients of European descent (i.e., Italian population), we utilizes patients' phenotypic information age, gender, and COVID-19 severity grading classification (0 - 5). To perform the downstream analyses, we further binarized the patients' COVID-19 outcome severity grading classification 5, 4, 3 were grouped as severe and coded as 1 versus grading 0 grouped as asymptomatic and coded as 0. The age distribution of the participants ranges from 18 Years and older (Adult, Older Adult). The Gender this study considered were all sexes. Patients' genotypic information contains a reference (Ref) or alternative (Alt) alleles in either severe or control groups which were defined by employing an additive model, whereby homozygous genotype (1/1) has twice the risk (or protection) of the heterozygous type (0/1 or 1/0).
Recruitment	The eligible criteria used to enroll patients for the study were 18 years and older (Adult, Older Adult) and SARS-CoV-2 PCR positive swabs from a study population of hospitalized patients, outpatients, and asymptomatic individuals. Healthy volunteers were, however, denied participation in this study. The GEN-COVID Multicenter Study group coordinated the enrollment of the patients used for this study via an observational study designed which collected and systematized the biological samples and clinical data across multiple hospitals and healthcare facilities in Italy to derive patient-level phenotypic and genotypic data.
Ethics oversight	This study was approved by the ethical committee at the COVID-19 Biobank of Siena, which is part of the Genetic Biobank of Siena, member of BBMRI-IT, of Telethon Network of Genetic Biobanks (project no. GTB18001), of EuroBioBank, and RD-Connect.

Note that full information on the approval of the study protocol must also be provided in the manuscript.

Clinical data

Policy information about clinical studies

All manuscripts should comply with the ICMJE guidelines for publication of clinical research and a completed CONSORT checklist must be included with all submissions.

Clinical trial registration	NCT04549831
Study protocol	The full trial protocol can be accessed via (https://clinicaltrials.gov/ct2/show/NCT04549831)
Data collection	The patients' genetic WES and clinical datasets were collected from SARS-CoV-2 positive individuals that were 18 years of age and above. The SARS-CoV-2 PCR positive samples of the patients were collected on a swab and performed massively parallel sequencing of the host genome. The biological samples and clinical data were collected across multiple hospitals and healthcare facilities in Italy. The Genome-Wide Association Studies was performed by the Institute of Molecular Medicine in Finland (FIMM), while the Whole Exome Sequencing (WES) was performed by the University of Siena. The study enrolled 2000 cohort participants of European descent (Italian population) at the beginning of April 2020 and the study is expected to terminate by the beginning of April 2026.
Outcomes	1) Primary outcome measure: To identify the genetic determinants of COVID-19 severity<br>Identification of one or more candidate gene(s) responsible for the severe outcome and subsequent use of it/them for prognostic purposes and preventive treatment and/or care.<br>How we achieved this measure:<br>In this study, we implored the use of an innovative 5-fold CV splitting strategy of the patients' phenotypic information (80% training and 20 % testing sets) and uses the odds ratio statistics and p-value approach to filter for significant variants from the corresponding patients' genotypic WES dataset information that constitute each of the training set splits, same variants information was used to curate the test sets for each of the 5-fold CV splits. These filtered significant variants were remapped using their alleles frequency counts and covariates (age and gender) to develop the feature matrices for each of the 5-fold CV splits. We used interpretable machine learning techniques for the classification task to train each of the 5-fold CV splits, unsupervised machine learning techniques for the clustering task of the non-zero weighted pool of variants identified using decision tree models (Random Forest and XGBoost classifiers) aggregated across the 5-fold CV splits, domain knowledge for pathway enrichment analysis, and Phenome-wide disease-variants association approach to analyze the pools of significant variants with non-zero. Our downstream analyses identified 16 fully supported variants (i.e., variants that received non-zero weights consistently from decision tree models across the 5-fold CV splits aggregated) 9 out of the 16 variants we identified coincide with variants identified and established from recent studies to linked with the severity of SARS-CoV-2 infections and coronavirus disease in patients. We also identified via the PCA clustering technique, a cluster of patients (29 in number with homogenous severity of 98 %) whose COVID-19 severity is most likely influenced by genetic interactions and lesser covariates influence (age and gender).<br><br>2) Secondary Outcome measures: to identify the genetic determinants of COVID-19 clinical trajectories.<br>Identification of candidate gene(s) responsible for the COVID-19 clinical trajectories.<br>How we achieved this measure:<br>We implored the Phenome-wide Association technique by leveraging the Bioinformatics tool of OpenTarget web tool (an open-source web browser Bioinformatics tool) to associate our identified variants (i.e., non-zero weight variants aggregated across the 5-fold splits CV from the decision tree models Random Forest and XGBoost classifiers) with disease traits which could lead to plausible clinical trajectories of the COVID-19 disease in patients.

Dual use research of concern

Policy information about dual use research of concern

Hazards

Could the accidental, deliberate or reckless misuse of agents or technologies generated in the work, or the application of information presented in the manuscript, pose a threat to:

No	Yes	
☒	☐	Public health
☒	☐	National security
☒	☐	Crops and/or livestock
☒	☐	Ecosystems
☒	☐	Any other significant area

Experiments of concern

Does the work involve any of these experiments of concern:

No	Yes	
☒	☐	Demonstrate how to render a vaccine ineffective
☒	☐	Confer resistance to therapeutically useful antibiotics or antiviral agents
☒	☐	Enhance the virulence of a pathogen or render a nonpathogen virulent
☒	☐	Increase transmissibility of a pathogen
☒	☐	Alter the host range of a pathogen
☒	☐	Enable evasion of diagnostic/detection modalities
☒	☐	Enable the weaponization of a biological agent or toxin
☒	☐	Any other potentially harmful combination of experiments and agents
